# Future innovations for the treatment of facial nerve paralysis

**DOI:** 10.1016/j.jpra.2025.05.009

**Published:** 2025-05-29

**Authors:** Andrea Weinzierl, Erica Piccinni, Sedef Kollarik, Stefania Konstantinidi, Yoan Civet, Yves Perriard, Pietro Giovanoli, Nicole Lindenblatt

**Affiliations:** a Department of Plastic Surgery and Hand Surgery, University Hospital Zurich, Raemistrasse 100, 8091, Zurich, Switzerland; b Faculty of Biomedical Sciences, Università della Svizzera Italiana (USI), Via Buffi 13, 6900, Lugano, Switzerland; c Integrated Actuators Laboratory, Ecole Polytechnique Fédérale de Lausanne, Rue de la Maladière 71b, 2002, Neuchâtel, Switzerland

**Keywords:** Facial nerve palsy, Facial nerve paralysis, Artificial muscles, Facial reanimation

## Abstract

Facial nerve injury and paralysis causes negative physical and psychosocial effects for afflicted patients. Impaired respiration, articulation, oral competence, as well as insufficient protection of the corneas may cause a markedly decreased quality of life and social isolation. The optimal management of underlying pathologies and the resulting symptoms is thus an ongoing topic of clinical and pre-clinical research and the optimal management of facial nerve injury is continuing to evolve. Recent technological advances have opened up exciting possibilities in this complex field, giving hope to treating physicians and afflicted patients alike. The present narrative review summarizes several concepts regarding biomedical engineering and facial reanimation with an overview of the current literature.

## Introduction

Facial nerve paralysis can result from various medical conditions including genetic anomalies, infection, cholesteatoma, trauma, malignancy, autoimmune problems, and pregnancy.^1^ It may also occur as a complication of surgeries in the head and neck region.^2^ Facial nerve injury results in aesthetic, functional and emotional disturbances for the affected patients, especially since any attempt to smile aggravates the facial asymmetry.^3^^,^^4^ The functional deficit of the muscles innervated by the facial nerve negatively affects respiration, articulation, oral competence, as well as protection of the corneas by sufficient eyelid closure, resulting in a drastically decreased quality of life.

Based on the cause, duration, severity and whether it is present in one or both sides of the face, treatment options vary. Current standard treatments for facial nerve paralysis can be categorized based on the stage and severity of the condition, including early versus late, and partial versus complete paralysis. In the early stages, standard treatments include physiotherapy and surgical interventions such as nerve grafts or nerve transfers. In late-stage cases, multistage or single-stage muscle transfers are commonly employed to restore facial function. In addition, botox injections may be used to improve facial symmetry by elimination synkinesis of the affected side or decreasing facial muscle movement of the healthy side.

Some treatment options are very time sensitive, as long-term facial palsy results in the degeneration of the distal nerve segment and muscle atrophy.^5^ Surgical treatment is used in patients with a complete disruption of nerve continuity, ideally restoring nerve continuity. Moreover, reconstructive surgical procedures may be considered for patients who are unlikely to achieve satisfactory recovery with observation and/or pharmacological treatment. Ideally, re-innervation of the muscle must be achieved within 12 to 18 months of initial injury. In patients that present with longer ongoing facial palsy, complex secondary facial reanimation procedures may be required to restore symmetry and voluntary facial movement.^6^ However, even after surgical treatment with procedures such as free muscle graft or myoplasty, the recovery is slow and often incomplete or even unsuccessful in many cases. Moreover, even after movement is restored, the affected side is often weaker than the unaffected side of the face. Facial nerve palsy thus remains a subject of both clinical and preclinical research, as its management and therapy continue to evolve. Innovative strategies emerge and give hope to afflicted patients, as well as physicians treating this condition, based on the type of underlying condition and necessary facial reanimation.

Thus, the objective of this review is to provide a short overview of several groundbreaking strategies currently pursued in clinical and preclinical research. The review was conducted based on an online literature search in the MEDLINE and Scopus database.

## Neural tissue engineering / bioengineering

Injury to peripheral nerves poses a significant challenge to restoring nerve function. Successful recovery of facial nerve function relies on the outgrowth of new axons, proper myelinization, and accurate reinnervation of the target tissues. Traditional surgical techniques include nerve-coaptation or the use of collagen tubes, cadaveric nerve allografts, polyglycolic acid conduits or donor nerves. However, challenges such as a diminished autograft performance due to modality and fascicular mismatch when using a predominantly sensory nerve (e.g., sural) to repair the facial nerve as a predominantly motor nerve, remain.^7^ New data on nerve transection repair, nerve gap injuries and the application of nerve conduits and adhesives have led to a better understanding of nerve regeneration. As a result, significant advances in the field of tissue engineering have been achieved.

Neural tissue engineering tends to focus on three important challenges in order to guide nerve regeneration and prevent nerve tissue dislocation: cell cultivation (Schwann cells (SC), mesenchymal stromal cells (MSC) and/or neural stem cells (NSC)), the use of neurotrophic factors and the creation of optimal artificial neural rooms.^8^ MSC-based therapies have shown promise for facial nerve injury, with studies demonstrating their ability to differentiate into SC-like cells and improve axon regeneration.^9^^,^^10^ MSC can be sourced from different tissues such as bone marrow,^11^ adipose tissue,^12^^,^^13^ gingiva^14^ and dental pulp.^15^^,^^16^ Promising results with this approach have been achieved in several preclinical studies.^17^, ^18^, ^19^ Interestingly, one effect of the presence of stem cells is the direct delivery of neurotrophic factors.^20^ The presence of these factors plays a crucial role in maintaining the microenvironment necessary for nerve fiber regeneration when peripheral nerve injury occurs.^21^ They help support the survival and growth of nerve cells by promoting the repair process, stimulating axonal growth, and encouraging the differentiation of SCs. Thus, besides a topical application, e.g. via hydrogels, they may be loaded onto nerve scaffolds or conduits to improve regeneration.^22^ Ideally, a gradual and consistent release over time should be achieved. Among others, beneficial effects have been shown for insulin-like growth factor (IGF)-1,^23^ fibroblast growth factor (FGF),^24^ hepatocyte growth factor (HGF)^25^ and neuregulin-1.^26^ Conduits may simultaneously deliver multiple growth factors, optimizing nerve regeneration. For instance, using vascular endothelial growth factor (VEGF) and IGF-1 in a murine model has improved muscle motor function and nerve repair.^27^ Human placental extract has also been used successfully for facial nerve regeneration in rats, as it contains a combination of epidermal growth factor, FGF, IGF, transforming growth factor, VEGF and HGF.^28^

Recent data demonstrates that the morphological characteristics and orientation effect of scaffolds and conduits play a central role in the regulation of cell differentiation, proliferation and migration.^29^ The ideal scaffold materials for tissue engineering should exhibit several key properties to support effective facial nerve regeneration. High biocompatibility helps minimizing the immune rejection, promotes healing, and reduces scarring. Potential materials should have appropriate porosity and selective permeability to facilitate optimal cell adhesion, nutrient and oxygen delivery, and waste removal.^30^ In addition, topological features such as fine, directional surfaces enhance the microenvironment at the injury site, preventing nerve dislocation and encouraging the guided growth of cells and tissue.^31^^,^^32^ Besides a growing knowledge regarding the used materials, the fabrication of neural constructs has benefitted heavily form the technical advances in recent years, allowing for an increasingly close recreation of the physicochemical and biological characteristics of natural extracellular matrix. Approaches include electrospinning or 3 dimensional (3D) bioprinting. Due to the controllable porosity, high surface-to-volume ratio and suitable mechanical properties of electrospun materials, it is possible to fabricate fibers with various orientations and layered structures. This technique has also been used to create facial nerve conduits.^33^^,^^34^ Similarly, bioprinted nerve constructs promoted regeneration and functional recovery in rat facial nerves when used to bridge segmental defects.^35^ This concept is particularly interesting, as it would allow for personalized conduits tailored to each patient’s needs, e.g., allowing bifurcated or irregular-shaped conduits.^36^^,^^37^ If future research is successful in translating these findings into human patients, neural tissue engineering may be able to increase the chances for successful facial nerve regeneration and improve surgical outcomes.

## Electrical stimulation

Traditionally, electrical stimulation has been used to improve facial function through non-invasive activation of muscles.^38^ However, direct electrical stimulation of muscles no longer innervated by the facial nerve, such as the orbicularis oculi muscle, is also a potential therapeutic tool for inducing eye closure in individuals suffering from facial nerve palsy. Achieving effective and pain-free lid closure may be able to prevent complications like corneal exposure or keratopathy. Candidates for orbicularis oculi stimulation should have intact, not yet atrophied muscle tissue, even if partially denervated. Notably, isolated cases have been described, where stimulation was able to increase movement in clinically completely paretic muscles years after the onset of facial nerve palsy, provided that the muscle was not totally denervated.^39^

To achieve full functionality and a natural-looking eye blink, several electrical stimulation parameters need to be considered including pulse mode. Complete eyelid closure can be achieved through single-pulse^40^^,^^41^ or the pulse-train electrical stimulation,^42^^,^^43^ with the latter seemingly more effective and requiring lower current intensities, thereby reducing patient discomfort.^43^ Adjusting pulse width affects lid closure efficiency, though excessively long pulses can interfere with muscle activation due to conflicting stimulus phases. Sachs et al.^41^ has demonstrated in a rabbit model that 10 pulses per train seem to achieve optimal results, likely through wave summation and tetanic contraction.^43^ It is important to consider, that pain perception increases with higher stimulation amplitudes, but can be mitigated using pulse-train stimulation and optimizing frequency settings.

To facilitate eye closure, electrodes are typically placed along the zygomatic branch of the facial nerve or directly over the orbicularis oculi muscle.^39^^,^^44^^,^^45^ The delicate nature of the orbicularis oculi muscle presents a challenge for electrode placement, as there is a risk of missing the target area entirely. Additionally, complications such as bleeding or edema at the implantation site can further hinder stimulation by disrupting electrode contact or altering tissue conductivity. In fact, even a higher fat content may hinder stimulation, as shown by a positive correlation between higher BMI values and electric stimulation amplitudes for forehead and cheek movements.^45^ Placement strategy also influences the effectiveness of stimulation, with horizontal electrode arrays along muscle fibers yielding better results.^46^ Animal studies suggest that multiple-channel stimulation requires lower intensities and achieves more natural closure compared to single-field stimulation.^47^ These factors highlight the importance of precise electrode placement and careful monitoring to optimize outcomes in facial nerve stimulation and functional recovery.

In an ideal pacing device, a closed-loop system with an input signal from the healthy side generates an output signal for stimulating the affected orbicularis oculi. This typically involves detecting muscle activity on the healthy side to trigger stimulation on the affected side using electromyography (EMG).^44^^,^^46^^,^^48^^,^^49^ This method can however be affected by signals from nearby muscles like the zygomaticus and masseter, causing unintended blinking.^48^ Therefore, additional software to filter out non-orbicularis oculi signals may be necessary.^48^^,^^50^ Alternative approaches for blink detection include using a gyroscope fixed to the contralateral, healthy eyelid^43^ and infrared-equipped glasses,^51^ though latency issues persist.

So far, research has mostly explored this therapeutic concept in preclinical animal models.^41^^,^^46^^,^^52^^,^^53^ For instance, Zhang et al. successfully performed implantation of an artificial facial nerve to restore orbicularis oculi muscle function in rabbits with unilateral peripheral facial paralysis.^54^ Interestingly, several feasibility studies in humans already exist as well.^48^^,^^49^^,^^55^^,^^56^ Cervera-Negueruela et al.^57^ have recently published initial data on a completely wearable device that was able to significantly improve overall eye closure, as well as cornea coverage. It is therefore tempting to speculate, that facial pacing may be used as a non-permanent and temporary solution for afflicted patients in the future. Thought further research in this field is warranted to exclude possible negative effects of facial pacing such as a possible induction of synkinesis, a concern that has been raised in the past.^45^ Moreover, rare complications like vasospasm after the implantation of neurostimulation devices have been reported in the past and may also play a role for facial pacing.^58^ However, if future research is successful in refining stimulation parameters, assessing long-term safety, and determining ideal electrode placement, facial pacing may represent a valid treatment option, e.g., for patients unfit or unwilling to undergo surgical interventions.

## Artificial muscles for facial reanimation

In cases where nerve coaptation cannot be performed prior to muscle atrophy or due to patient specific factors, e.g., a congenital defect as the underlying condition, patients often require complex surgical treatment such as multi step reconstruction with cross-face nerve grafting and subsequent free muscle grafting. The development of an implantable artificial muscle may present an alternative way of facial reanimation for patients who are unable or unwilling to face this type of extensive and often multi-step surgery. Moreover, they may represent a possibility to reconstruct facial movement without any donor site morbidity.

Biological muscle fibers exhibit quick reaction times with large linear forces, while still being small in volume. A variety of actuators (motion-generating devices) have been proposed in the field of robotics, including electromagnetic motors, piezoelectric ceramics or shape memory alloys. However, due to their poor biocompatibility, their use in human tissue is limited. However, newer technologies may be able to overcome this obstacle.^59^ Artificial muscles have evolved rapidly in recent years. Over time, problems including limited cycle life, inefficient energy conversion, high costs and hysteresis have been investigated, bringing them closer to practical clinical use and making them a possible approach to reconstruct muscle movement.^60^^,^^61^

So far, implantable devices have mostly been suggested to enable eye closure, though smile reanimation may also be possible in the future. While currently only lid loading with gold or platinum weights is being practiced in clinical routines, implantable prosthesis to completely restore a dynamic blink have been suggested.^62^, ^63^, ^64^, ^65^ These devices create eyelid closure by means of an artificial sphincter mechanism, or eyelid-sling. Hasmat et al.^62^ have presented work on an electromagnetic actuator connected to a tendon sling with promising results in cadavers. In this study, the generated magnetic field creates lateral movement in an electromagnet that is translated to the tendon sling, and ultimately the eyelid. Senders et al.^63^ applied this sling mechanism in a cadaver study using a device based in electro-active polymers. Moreover, a preclinical follow up study in a gerbil model has explored the durability and biocompatibility and has achieved activating motion with minimal fibrous capsule surrounding the implants.^66^ These thin and pliable dielectric elastomer actuators (DEAs) seem to be a particularly promising candidate for facial reanimation, as their flexibility and power-to-weight ratio compares favorably with natural muscle.^67^, ^68^, ^69^, ^70^, ^71^, ^72^ The DEAs soft and elastic material opens up new possibilities and may make it a suitable option for facial reanimation, overcoming challenges such as the comparatively thin soft tissue coverage of the face and the need for high precision due to the symmetrical movements of the contralateral face.^73^, ^74^, ^75^ Initial promising concept papers regarding smile reanimation and blink restoration using DEAs exist, where this technology was used for instance to create visible displacement of a skin model synchronous with a healthy participant's mouth equipped with surface EMG electrodes.^74^^,^^75^

Facial reanimation by means of artificial muscles is a technology that is still in its infancy, though rapid progress raises hope for a future implementation into clinical routines. Advancements in engineering must address challenges such as developing a suitable sized internal power source for implantation. Additionally, further research is warranted to explore long-term durability and reproducibility of the movement created in vivo by these devices. However, initial proof of concept studies have shown that artificial facial reanimation may be possible in the foreseeable future.^62^, ^63^, ^64^, ^65^, ^66^

## Discussion

**Figure 1:**
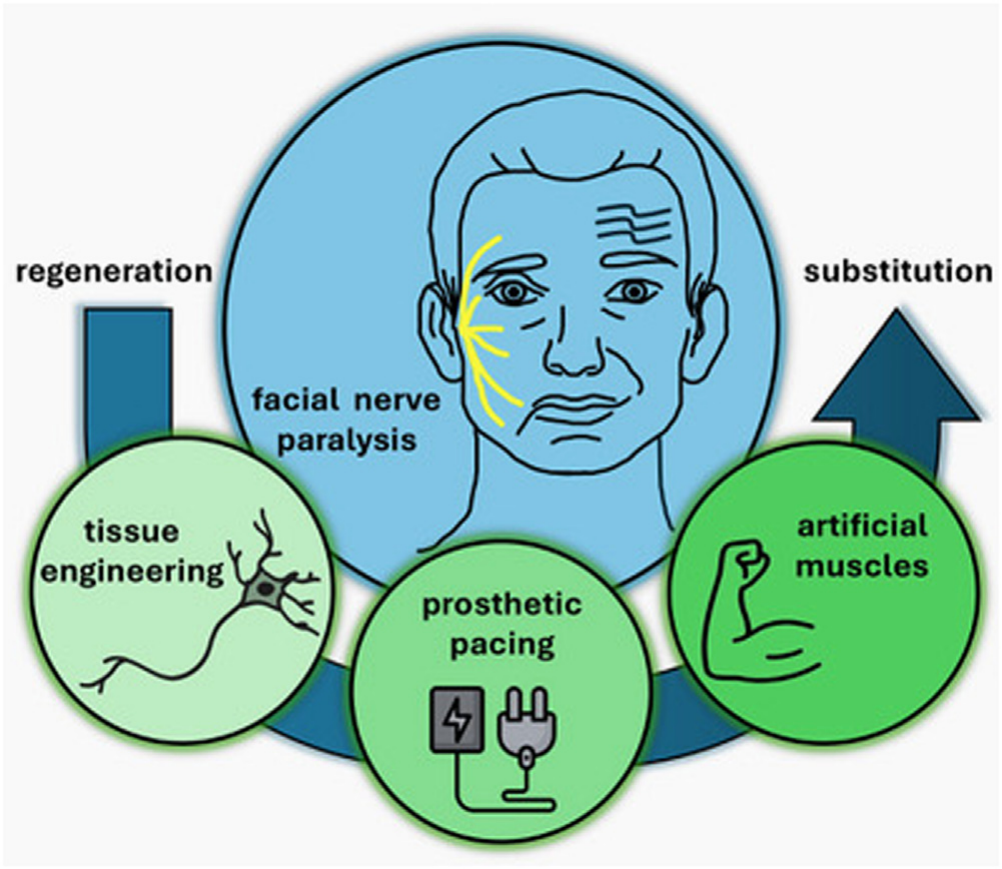
Current and future innovations may provide afflicted patients with treatment options spanning from regenerative approaches to a substitution of denervated muscles to manage facial nerve paralysis.

Facial nerve paralysis, regardless of its underlying cause, represents a profound challenge for both the affected individual and the medical community. The intricate nature of the facial nerve's role in motor control for facial expressions, protection of the eyes, and other vital functions underlines the necessity of effective and timely treatments. Although traditional methods, such as surgical interventions and pharmacological management, have provided some success in restoring facial function, the limitations of these therapies - such as the slow recovery and the persistence of facial asymmetry - prompt the exploration of innovative strategies. This review highlights groundbreaking approaches in tissue engineering, electrical stimulation, and artificial muscle development, all of which hold significant promise for improving the treatment of facial nerve paralysis (Figure 1).

The field of tissue engineering has made substantial advancements in the context of facial nerve repair. New research may offer more sophisticated alternatives to traditional co-adaptation or the traditionally used autografts and nerve conduits in the future. Electrical stimulation has emerged as a second promising option for improving facial function, particularly in restoring eye closure. Early studies have demonstrated that electrical stimulation can help induce eyelid closure and protect the cornea from exposure. Wearable devices that deliver electrical stimulation to the orbicularis oculi muscle, significantly improving eyelid closure and offering a non-invasive option for patients with facial nerve paralysis seems to be a particularly promising development in this field. Lastly, the development of artificial muscles for facial reanimation presents an exciting new frontier in facial nerve paralysis treatment. For patients who have experienced muscle atrophy due to delayed nerve repair or congenital defects, artificial muscles could provide a solution that bypasses the need to sacrifice healthy tissue for nerve and muscle grafts. Interestingly, the strategies discussed in the present review thus seem to encompass the entire spectrum from regeneration of the facial nerve up to a functional substitution by means of artificial muscles. Such a “bionic face” long seemed unimaginable due to the intricate movements of facial muscles. The term “bionic” generally refers to devices that establish a direct connection with the remaining nervous or muscular system of individuals with impairments and was initially popularized in the 1970ies by the US Tv show “The Six Million Dollar Man and Bionic Woman”, in which superpowers were imparted through electromechanical implants.^76^ Though the implantation of artificial muscles for facial reanimation may yet seem futuristic, recent technological advancements have brought a bionic face within reaching distance.

## Conclusion

The management of facial nerve paralysis remains a complex clinical challenge, but recent advances in tissue engineering, electrical stimulation, and artificial muscle development offer hope for more effective treatments in various clinical scenarios. While current treatments for facial nerve paralysis exist, they do not come without certain drawbacks. Thus, the rapid pace of research in this field offers hope for even more evolved treatments in the future, both regarding functional recovery and quality of life. Future clinical trials and longitudinal studies will be essential in validating these approaches and ensuring that the benefits of these technologies can be fully realized.

## Funding

Parts of the scientific work cited in this review have been funded by the Werner Siemens Foundation.

## Ethical approval

Not required.

## Declaration of competing interest

Nicole Lindenblatt acts as consultant and scientific advisor for Medical Microinstruments (MMI). All remaining authors have declared no conflicts of interest.
